# Indicators of Good Nursing Practices for Vulnerable Groups in Primary Health Care: A Scoping Review*

**DOI:** 10.1590/1518-8345.5203.3488

**Published:** 2021-10-29

**Authors:** Emiko Yoshikawa Egry, Lucimara Fabiana Fornari, Monica Taminato, Sônia Maria Garcia Vigeta, Rosa Maria Godoy Serpa da Fonseca

**Affiliations:** 1 Universidade de São Paulo, Escola de Enfermagem, São Paulo, SP, Brazil.; 2 Universidade Federal de São Paulo, Escola Paulista de Enfermagem, São Paulo, SP, Brazil.; 3 Scholarship holder at the Conselho Nacional de Desenvolvimento Científico e Tecnológico (CNPq), Brazil.; 4 Scholarship holder at the Coordenação de Aperfeiçoamento de Pessoal de Nível Superior (CAPES), Brazil.

**Keywords:** Community Health Status Indicators, Vulnerable Populations, Primary Health Care, Nursing, Review, Qualitative Research, Indicadores de Salud Comunitaria, Poblaciones Vulnerables, Atención Primaria de Salud, Enfermería, Revisión, Investigación Cualitativa, Indicadores de Saúde Comunitária, Populações Vulneráveis, Atenção Primária em Saúde, Enfermagem, Revisão, Pesquisa Qualitativa

## Abstract

**Objective:**

to map the indicators of Good Nursing Practices in Primary Health Care, from the perspective of Collective Health, reported to the vulnerable social groups.

**Method:**

this is a scoping review according to the PRISMA Extension for Scoping Reviews. The searches were carried out in2020 in six databases and in a virtual library. Independent reviewers performed the reading of the full texts, as well as treatment, analysis and synthesis of the content.

**Results:**

a total of 13 articles were found, the first from 2007 and the last from 2020. The data were classified according to the following empirical categories: assessment and control of health conditions(3 indicators); assessment of knowledge about health(3 indicators); use of sociodemographic characteristics to estimate risks or vulnerabilities(3 indicators); assessment and monitoring of health needs(5 indicators); promotion of safety and trust in health services(6 indicators); and assessment of the care process(4 indicators).

**Conclusion:**

the articles showed a variety of indicators that assess the interventions carried out in the context of Nursing in Primary Care with vulnerable social groups. These indicators are related to health conditions, especially those of the biopsychological body, reported to vulnerable populations, especially women, children, adolescents and older adults.

## Abstract (es)

**Objetivo:**

mapear los indicadores de Buenas Prácticas de Enfermería en la Atención Primaria de la Salud, desde la perspectiva de la Salud Colectiva, en los grupos sociales vulnerables.

**Método:**

revisión de alcance de acuerdo con PRISMA *Extension for Scoping Reviews*. En 2020 se realizaron búsquedas en seis bases de datos y una biblioteca virtual. Revisores independientes realizaron la lectura de los textos completos, el tratamiento, el análisis y la síntesis del contenido.

**Resultados:**

se encontraron 13 artículos, el primero de 2007 y el último de 2020. Los datos se clasificaron según las categorías empíricas: evaluación y control de las condiciones de salud (3 indicadores); evaluación del conocimiento sobre la salud (3 indicadores); uso de características sociodemográficas para estimar riesgos o vulnerabilidades (3 indicadores); evaluación y seguimiento de las necesidades de salud (5 indicadores); promoción de la seguridad y la confianza en los servicios de salud (6 indicadores); evaluación del proceso de atención (4 indicadores).

**Conclusión:**

los artículos mostraron una variedad de indicadores que evalúan las intervenciones realizadas en el contexto de la Enfermería en la Atención Primaria con grupos sociales vulnerables. Estos indicadores están relacionados con las condiciones de salud, especialmente del cuerpo biopsíquico, de las poblaciones vulnerables, especialmente mujeres, niños, adolescentes y adultos mayores.

## Abstract (pt)

**Objetivo:**

mapear os indicadores de Boas Práticas de Enfermagem na Atenção Primária em Saúde, na perspectiva da Saúde Coletiva, reportados aos grupos sociais vulneráveis.

**Método:**

trata-se de uma revisão de escopo conforme o PRISMA *Extension for Scoping Reviews*. As buscas foram realizadas no ano de 2020 em seis bases de dados e em uma biblioteca virtual. Revisores independentes realizaram a leitura dos textos completos, o tratamento, a análise e a síntese do conteúdo.

**Resultados:**

foram encontrados 13 artigos, sendo o primeiro de 2007 e o último de 2020. Os dados foram classificados segundo as categorias empíricas: avaliação e controle das condições de saúde (três indicadores); avaliação do conhecimento sobre saúde (três indicadores); uso de características sociodemográficas para estimar riscos ou vulnerabilidades (três indicadores); avaliação e acompanhamento das necessidades em saúde (cinco indicadores); promoção da segurança e confiança nos serviços de saúde (seis indicadores); avaliação do processo de cuidar (quatro indicadores).

**Conclusão:**

os artigos mostraram uma variedade de indicadores que avaliam as intervenções realizadas no âmbito da Enfermagem na Atenção Primária junto aos grupos sociais vulneráveis. Esses indicadores têm relação com as condições de saúde, principalmente do corpo biopsíquico, reportadas às populações vulneráveis, destacando-se mulheres, crianças, adolescentes e idosos.

## Introduction

The concept of Good Practices in the health area is broad and diversified. A study defines it as the best way to identify, evaluate and implement information through monitoring the health care results^(1)^. Another study considers it as a technique or methodology with proven reliability to guide a given result^(2)^. For other authors, it corresponds to the triad made up by the best results of scientific research studies, clinical knowledge and the users’ needs^(3)^.

With regard to Nursing, the concept of Good Practices is understood as the critical process of reflection on the actions taken, in search for the effectiveness of a practice. Knowing the meaning of the practice is essential because, based on this knowledge, the nurse can apply the necessary amount of intellect in care organization. In addition to that, the understanding of best practices is based on the assumption that, in a given context, some solutions are superior for solving problems when compared to others^(4)^.

From the perspective of Collective Health Nursing, it is considered that Good Nursing Practices(GNPs) in Primary Health Care(PHC) must contain principles such as: observing that this field of practice takes place in the geopolitical territory of social production and reproduction and that work in health aims at transforming the population’s epidemiological profiles^(5)^. It is in the territory that the social phenomena expressed in the population’s health profiles manifest themselves explicitly and demand knowledge and competences from nurses to recognize health needs and to face the vulnerabilities to which different population groups are exposed^(6)^.

Given the diversity of concepts of Good Practices, it is considered that, in addition to implementing them, it is necessary to establish criteria that may support the construction of indicators in order to parameterize care and the actions resulting from it. Indicators are quantitative or qualitative parameters that detail the objectives of a proposal according to its conduction(evaluation of the process) or scope(evaluation of results). In addition to that, they point to trends and act as instruments that do not operate by themselves^(7)^.

Although GNPs are found in the scope of PHC, studies on indicators that support these practices are not sufficiently known. Given this, the scientific question of this study was the following: What indicators are used to support the GNPs reported to vulnerable social groups in PHC? Based on this, the objective of this study was to map the indicators of GNPs in PHC, from the perspective of Collective Health, reported to vulnerable social groups.

## Method

This is a scoping review following the recommendations of the Preferred Reporting Items for Systematic Reviews and Meta-Analyses Extension for Scoping Reviews(PRISMA-ScR). This type of review is used to map evidence, explore the breadth or extension of the literature, and inform future research studies. It is also recommended to identify and analyze knowledge gaps about a particular research topic or field^(8)^.

The protocol for this scoping review is registered in Open Science. The review question was elaborated through the PCC strategy, which advocates the following mnemonic acronym as fundamental elements: P-Population, C-Concept and C-Context. For the search of evidence, the following elements were defined: P-Vulnerable social groups, C- Indicators of Good Nursing Practices and C-Primary Health Care. Therefore, the review question adopted was: What indicators are used to support the Good Nursing Practices reported to vulnerable social groups in Primary Health Care?

The eligibility criteria were studies published in English, Spanish and Portuguese, with no restriction regarding publication date. Primary, empirical, quantitative and qualitative studies with any design or methodology were included; as well as studies that pointed out indicators or means of evaluating a GNP in PHC regarding vulnerable social groups, studies on the health assessment of the vulnerable population resulting from some intervention(policy or practice), and studies on practice or evaluation from the point of view of changing the health profile or pre-existing condition. Studies related to the professionals’ perspective on the practice or effectiveness of the practice in PHC regarding vulnerable social groups were excluded, as this perspective is expressed as an opinion and not as an indicator.

Data collection was carried out in the databases that presented a multidisciplinary interface on the GNP phenomenon in PHC. The databases consulted were the following: Medical Literature Analysis and Retrieval System Online via PubMed(MEDLINE/PubMed), *Literatura Latino-Americana e do Caribe em Ciências da Saúde*(LILACS), PsycINFO, Cumulative Index to Nursing and Allied Health Literature(CINAHL), Scopus and Excerpta Medica Database(EMBASE). The Scientific Electronic Library Online(SciELO) was also accessed as an additional source. A manual search of the references of the primary and secondary studies identified in the electronic search was performed.

**Figure 1:**
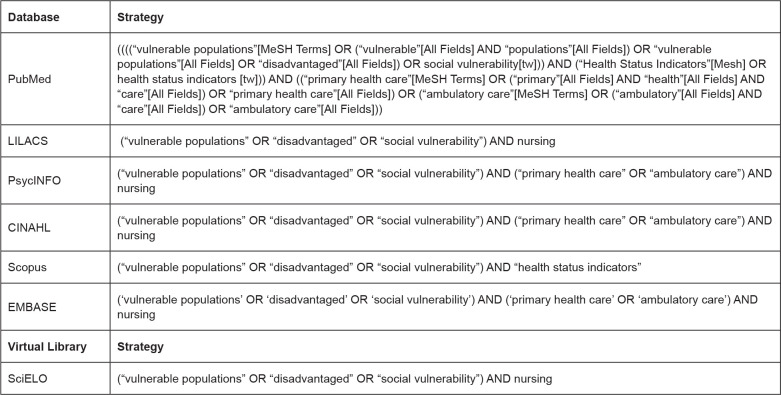
Database search strategies with boolean operators. São Paulo, Brazil,2020

The search strategies developed and used for each electronic database are shown in Figure 1 and were carried out in August2020, with no restriction regarding languages or publication means.

The study selection process was carried out by three independent reviewers and the differences were solved by a fourth reviewer.

**Figure 2:**
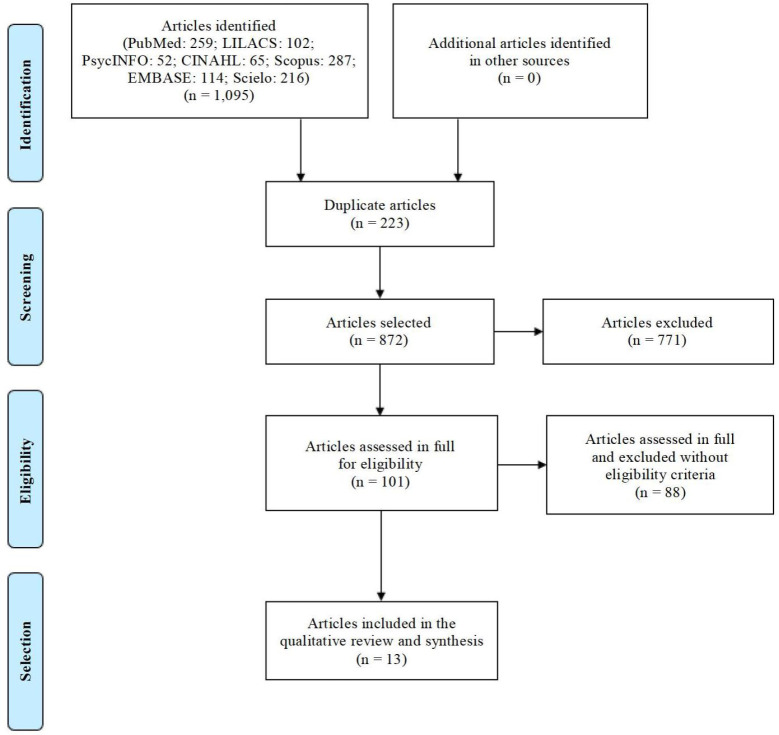
Preferred Reporting Items for Systematic Reviews and Meta-Analyses Extension for Scoping Reviews(PRISMA-SCR) flowchart on the selection of studies. São Paulo, Brazil, 2020

The selection of studies was carried out in two stages. In the first stage, the titles and abstracts of the references identified through the search strategy were evaluated; and the potentially eligible studies were pre-selected. In the second stage, the full texts of the pre-selected studies were evaluated in order to confirm their eligibility(Figure 2).

The selection of studies according to title and abstract was performed using the RayyanQCR^(9)^ digital tool, and the articles selected in each database were imported in the BibTex file format. Subsequently, three reviewers independently and blindly read the titles and abstracts in order to reduce the possibility of interpretive bias. Then, a fourth reviewer proceeded with the evaluation of the articles that presented divergences in order to include them or not in the study. In cases where the doubt about selection remained, the next stage was initiated, corresponding to the full-reading.

Data extraction from the full articles was performed using an instrument containing the following items: year of publication, concentration area, country where the article was produced, type of study, studied population, study locus, action performed and quality indicator. In addition to that, the following categories of analysis were considered: Social Determination of the Health-Disease Process, Health Needs, and Vulnerability and Care Process.

The Social Determination of the Health-Disease Process is associated with the understanding that health and disease result from people’s way of life, as a consequence of their insertion in the social production system^(10)^. Health Needs are linked to the potential to produce a health-genic paradigm, extrapolating needs. Vulnerability, that is, the fragility to face the vicissitudes of life, is related to the process of social exclusion and its confrontation with subjects and social groups^(11)^. The Care Process is based on the dynamics of the practical realization of the epistemic care object, prioritizing the social groups’ health needs^(12)^.

In data treatment, only peer-reviewed publications were considered. A critical evaluation of the texts was also carried out, mainly with regard to the methodology, according to the reviewers’ expertise.

The instrument used to collect the information was incorporated into the webQDA qualitative analysis software^(13)^. The characterization of the studies was carried out using descriptive codes. Descriptive coding was performed using the automatic encoding tool, which allows importing files in XML format. Subsequently, the data were coded by the Tree Code System, allowing for the emergence of the empirical categories through the thematic content analysis technique^(14)^, which enabled the elaboration of the knowledge syntheses. The exact considerations of the authors were considered as “indicators”, regardless of the concept or purpose they served.

## Results

The search in the databases mapped 1,095 potentially eligible studies, with13 remaining in the final sample, as shown in Figure 2.

Regarding the characteristics of the 13 studies selected, the first was published in2007 and the others, discontinuously, until2020. The largest production occurred in2019, with four articles, followed by 2018, with three. The areas referred to were as follows: Nursing(n=4), Health(n=4) and other areas(Geriatrics, Public Health, Maternal and Child Health, Psychology and Global Health) with one article each.

The countries where the studies were produced were the following: United States of America(n=6), Australia(n=3), and Brazil, Canada, Ireland and Spain with one article each. All were published in English, 10 studies having a quantitative approach and three with a qualitative approach.

In relation to the population studied, seven studies were conducted with adults, five of which included only women and three referred to older adults. There was an article about children and another about adolescents.

The vulnerable social groups associated with the studied population were described as living in disadvantaged urban areas. The participating women were characterized as mothers, victims of intimate partner violence or vulnerable; the children were described as suffering from a chronic disease or at risk of violence; the older adults, as people with dementia or multimorbidities; and the adolescents, as victims of violence, living on the streets, drug-addicts or serving socio-educational measures. Only one study addressed the Aboriginal population and another, the African-American population.

The evaluation of the actions performed in the studies was carried out through questionnaires(n=7), interviews(n=6), focus group(n=3), home visits(n=2), documents(n=2) and scale(n=1). Some studies used more than one strategy to assess the actions.

**Figure 3:**
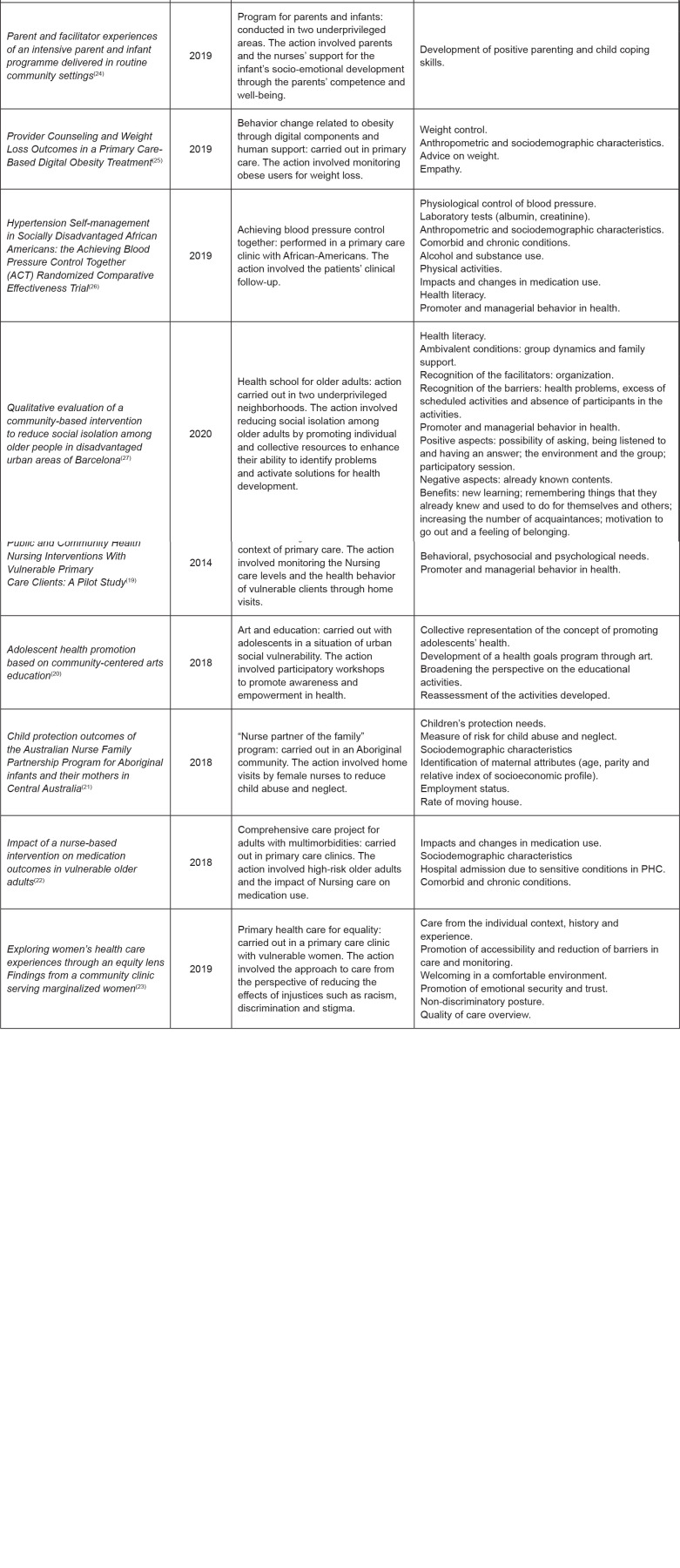
Characterization of the articles selected according to year of publication, action taken and indicators. São Paulo, Brazil, 2020

Figure 3 shows the characteristics of the publications according to the indicators.

**Figure 4:**
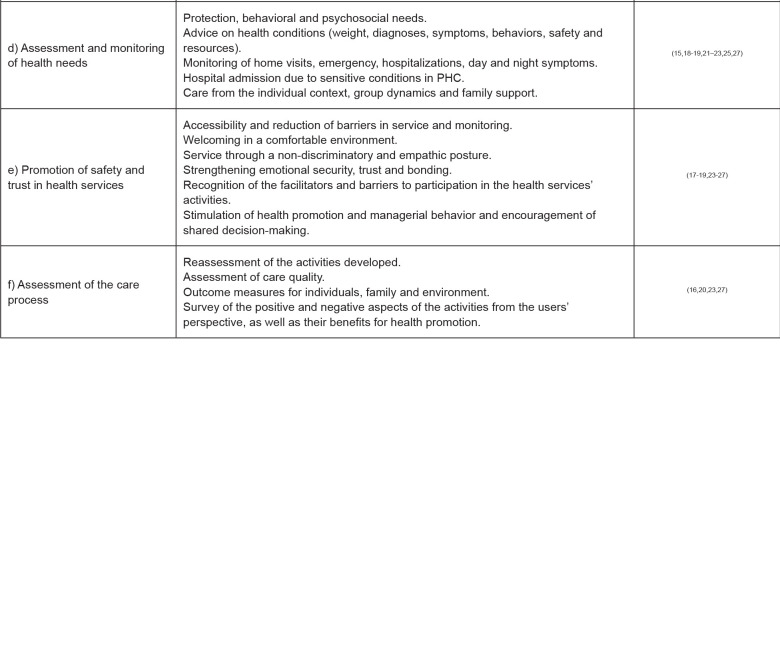
Distribution of the studies according to the emerging empirical categories and indicators. São Paulo, Brazil, 2020

The empirical categories that emerged from the scoping review were built from the selection of all the indicators listed in the selected articles. Although the research theme is different across the publications, it was possible to group the indicators according to the characteristics of the GNPs implemented and evaluated in the context of PHC. Figure 4 contains the indicators according to the empirical categories.

## Discussion

The knowledge of Collective Health Nursing has been developed from the deepening of the theoretical-methodological frameworks and the construction and testing of instruments aimed at analyzing the work processes with the potential to intervene in the objective reality and, therefore, in the health-disease process of different social groups. In addition to that, it is possible to verify an expansion of these instruments’ spectrum^(11)^.

This expansion was identified in the scoping review since, in 2018 and 2019, for example, a greater number of publications were presented when compared to the others in the last 13 years. In addition, it was verified that the instruments presented in the selected studies to analyze the Nursing actions have the capacity to support the use of indicators to assess the work process developed in the context of PHC.

Understanding the collective health Nursing work process emphasizes the concepts of social vulnerabilities and health needs as objects of the care practices. However, once the health needs are assessed, it is necessary to consider the challenge of recognizing and facing the social vulnerabilities, developing intervention actions and their respective assessments^(11)^.

The studies included in this review showed the range of people in a situation of social vulnerability assisted by Nursing. With regard to age, studies involving adults^(16-17,19,21,23-25)^, older adults^(18,22,27)^, children^(15)^ and adolescents^(20)^ stood out; regarding gender, there was predominance of studies involving women^(16-17,21,23-24)^; and, regarding race/ethnicity, of studies associated with the Aboriginal^(21)^ and African-American^(26)^ population.

The review showed that the Nursing work process developed with vulnerable populations can be evaluated through indicators that, for the most part, involve clinical health conditions. This aspect was evidenced in the first empirical category, in which the following are perceived as indicators: drug treatment, application of vaccines, diagnostic tests, anthropometric measures, comorbidities and chronic health problems.

A document produced by the American Nursing Association(ANA) assigns nurses the responsibility for the direct provision of care and the consequent results^(28)^. Although the document deals with evidence-based practices, it does not address GNP indicators in PHC.

The reduced number of studies included in this review shows how much GNP understanding can be offered in guides, but few studies prove support through indicators. Even so, Nursing shows its innovative face by acting on phenomena associated with vulnerabilities, such as ethnically subordinate populations in a given society or even generational groups such as adolescents belonging to subordinate social classes and older adults living in isolation at their homes. Furthermore, Nursing acted with populations that are barely visible, such as individuals who experience situations of intimate partner violence, which involves not only women but children who live in the same environment.

When talking about Nursing interventions in PHC aimed at vulnerable groups, those related to violence against women and children are certainly relevant. It was verified that few studies are produced with a view to seeking indicators of effectiveness or assessment of the results of the interventions. One of those found in this review shows the breadth, complexity and difficulties when it comes to producing indicators. MOSAIC(Mothers’ Advocates In the Community) describes a randomized clinical trial of support for mentor mothers to reduce intimate partner violence among pregnant women or new mothers. It is a broad and complex approach in which nurses’ actions are produced from different perspectives: evaluation of results, processes and economic impacts^(17)^.

The “Assessment and control of health conditions” empirical category aggregates GNP indicators in PHC to vulnerable social groups, linking them to health care. In this category, the family appears as a care object and, in this sense, the study carried out in Brazil^(29)^ could mean a leap in quality in terms of GNP indicators, as it validated an instrument capable of evaluating vulnerable groups both in relation to social and health conditions.

The second empirical category of the review also highlighted the importance of knowledge about health by the vulnerable populations, mainly through educational programs and activities. In this context, a Brazilian research study revealed the power of the theoretical framework based on the conception of awareness and empowerment. The intervention project involved participatory methodologies, such as workshops and models, collectively produced through artistic activities. In the end, the participants built a collective product that represented the concept of promoting adolescents’ health and encouraged self-determination for changes^(20)^.

In this same category, health literacy stood out, associated with the understanding of basic health information, so that users may support appropriate decision-making, with a view to promoting health care and preventing diseases. However, none of the articles that mention literacy used validated instruments for verification, as recommended^(30)^.

Users’ empowerment regarding knowledge about health represents a step forward in overcoming the hegemony of clinical-focused care and is extremely important for the theoretical framework of Collective Health Nursing, as it refers to the singular, particular and structural dimensions of the phenomena that affect the individuals or social groups that demand Nursing care^(10-11)^.

The third empirical category encompassed research studies using indicators related to sociodemographic characteristics to estimate social risks and vulnerabilities of the studied population^(21-22,25-26)^. Recognizing the risks and social vulnerabilities to which the population is exposed is important to equitably guide the care actions reported to social groups.

A study on racial inequality and mortality due to COVID-19 considered that social vulnerability allows understanding the unequal effects of the pandemic on the African-American population based on the social conditions and on exposure to risk. Different levels of poverty, segregation and discrimination influence the ability to respond to the disease. Therefore, the increase in social vulnerability is proportional to health inequality^(31)^.

The fourth category involves the assessment and monitoring of the health needs of vulnerable populations. The studies considered the protection, behavioral and psychosocial needs associated with sleep, weight and dementia. In addition to that, they took into account the individual context, life history, experience, and family support. The assessment of needs fulfillment was carried out through home visits and hospitalizations, mainly through Sensitive Conditions to Primary Health Care (SCPHC)^(15,18-19,21-23,25,27)^.

A study carried out to verify the effects of the intervention and the results in home-care through home visits found positive aspects corresponding to three domains: health management, general health promotion behavior, and physical activity subscale score. However, the authors recognize that delineating the specific effect of home visits performed by Nursing professionals in changing health behavior is complex, especially due to the difficulty of associating a particular strategy with a specific clinical result^(19)^.

The fifth category involved the users’ relationship with the health services, highlighting accessibility, welcoming, empathy, trust, non-discriminatory posture, strengthening the bond and recognizing the barriers that may influence the care and monitoring of health needs^(17-19,23-27)^.

A study carried out in Canada including 68 women with significant social and health inequalities showed the importance of the health team establishing a trusting relationship with the service users, particularly with those who had stigmatizing experiences or negative judgments when seeking the health services^(14)^. Based on this, it is considered that the use of indicators in PHC involves knowledge and the development of care oriented towards equality in health.

The action entitled Comprehensive Care for Multimorbid Adults Project(CC-MAP), evaluated through a controlled clinical trial and developed in Primary Care clinics of the Clait Health Systems, Israel’s largest insurer and integrated health provider, revealed that the oriented care model improved adherence to drug treatment and reflected in more attentive management to the health needs of vulnerable adults^(22)^.

In the sixth category, indicators that sought to assess the care process through the perspective about quality, outcome measures and the positive, negative and beneficial aspects of the interventions to meet health needs were included^(16,20,23,27)^.

One of the structured ways to assess the impact of Nursing actions in PHC and which attest to the GNPs in Collective Health could be the application of the Systematization of Nursing Care in Collective Health, subordinated to the International Classification of Nursing Practices in Collective Health(ICNPCH). Various studies produced in Brazil for the configuration of the Nursing diagnoses, interventions and outcomes could leverage the construction of indicators for the assessment of Nursing care in PHC^(32)^.

Finally, it is considered that valuing the Nursing work process involves measures of actions and quality of the care offered to the users-measures that will unveil problems associated with the scarcity of workforce and with the possibility of improving care^(33)^.

Nursing must also take ownership of common PHC tools, especially those that seek indicators for evaluating health policies, strategies and actions. A study analyzing instruments used in different countries found important domains in which the indicators must be adjusted or applied. Among them, the following stand out: national governance of gender inequality at the level of social protection and income inequality at the level of social protection; participation of civil society in the formulation of public policies, with emphasis on the indigenous and transgender population; and reorientation of the health sector towards the development of a basic set of indicators for governmental action aimed at improving equality in health^(34)^.

One of the study limitations was the *apriori* non-standardization of the indicators used by Nursing in the context of PHC, only mapping the existing ones. Future studies should be carried out to deepen and validate the indicators identified in this review. In addition to that, the review also presented the following limitations: the restricted number of selected databases, the data collection period, and the absence of a methodological evaluation of the articles included through a validated instrument. These limitations are justified due to the time taken to complete the review.

Despite these limitations, the results that emerged from the scoping review contribute to the advancement of scientific knowledge in the field of Collective Health Nursing, especially for the qualification of actions implemented in the context of PHC. The evidence mapped contributes to filling the knowledge gap about the indicators that underlie the GNPs, especially when reported to vulnerable social groups.

## Conclusion

The studies showed indicators that may qualify the interventions carried out in the context of Nursing with social groups which are considered vulnerable in PHC. With regard to these groups, residents in socially disadvantaged areas stood out, mainly involving the female population and the age groups corresponding to childhood, youth and older adults.

The indicators mapped also showed a relationship with the care of the biopsychological body beyond the multifactorial understanding of health-disease, entering the field of knowledge production for health promotion. In addition to that, they highlighted the nurses’ role in surveying the sociodemographic characteristics and health conditions, in monitoring health needs and in assessing the care process.
